# Modelling movement and stage-specific habitat preferences of a polyphagous insect pest

**DOI:** 10.1186/s40462-020-00198-7

**Published:** 2020-03-02

**Authors:** Adriano G. Garcia, Wesley A. C. Godoy, Fernando L. Cônsoli, Claudia P. Ferreira

**Affiliations:** 1 Department of Ecology, Evolution and Natural Resources, Rutgers – The State University of New Jersey, New Brunswick, 08901-8551 USA; 2 Department of Entomology and Acarology, University of São Paulo, ESALQ, USP, Piracicaba, 13418-900 Brazil; 3 IBB, UNESP, Botucatu, 18618-000 Brazil

**Keywords:** Parent-offspring conflict, Cellular automata, Crop calendar, Insect pest dynamics, Pest control

## Abstract

**Background:**

The feeding preferences of *Diabrotica speciosa* (Coleoptera: Chrysomelidae) cause a parent-offspring conflict, as providing the best host for the offspring development is detrimental to adult survival and fecundity. Understanding the implications of this conflict could help entomologists to implement pest-management programs. With this in mind, the foraging behaviour of *D. speciosa* was investigated using an individual-based model in two distinct scenarios.

**Methods:**

In an intercropping scenario, parent-offspring conflict was simulated when adult insects exploit two crops (corn and soybean) that provide different nutritional advantages for each insect stage. First, we compared three hypothetical types of adult dispersal, considering a continuous oviposition over time: diffusion, attracted to a fixed host and alternating the preference between hosts with frequency $\frac {1}{\tau }$, where *τ* is the time in days spent foraging for each host. We also simulated two principles: “mother knows best” (adult females foraging for corn during the oviposition period) and “optimal bad motherhood” (adult females remain foraging for soybean to maximise their own fitness during the oviposition period), but considering the existence of a pre-oviposition period. In a landscape scenario, we investigated the population dynamics in an area composed by 4 crop plots that change over time.

**Results:**

Among dispersal types considering continuous oviposition, the crop-alternating movement a-3 performed best, when close to an optimal *τ*. Additionally, *τ* was predicted to be influenced mainly by the width of crop rows. We also verified that the “mother knows best” strategy is better for the population growth than the “optimal bad motherhood”. In the landscape scenario, we observed that including fallow periods in the crop calendar and adopting a more-heterogeneous arrangement of crop plots reduced the density of this insect.

**Conclusion:**

Both the continuous and sequential oviposition simulations indicate that foraging involving switching of target crop benefits population fitness. In the landscape scenario, arranging crop plots more heterogeneously and avoiding vast areas of soybean can help farmers to control this insect pest. Additionally, fallow periods can also reduce significantly *D. speciosa* populations.

## Background

*Diabrotica speciosa* (Germar, 1824) (Coleoptera: Chrysomelidae) is a polyphagous insect pest occurring in South America, especially on corn (maize) and soybean crops in Brazil [1]. Adults feed on aerial parts of plants, whereas larvae consume roots and tubers [2]. Even small larval population densities are able to cause severe damage to corn plants, through decreasing the dry weight of the roots and upper part of the plant, and the plant height [3]. Multiple laboratory studies have examined the influence of different host plants on the development of larvae and adults, helping entomologists to clarify factors that drive the population dynamics of this insect in crop fields [4]. Corn and potato are suitable hosts for larval development of *D. speciosa*, whereas adults prefer feeding on leaves of potato and beans [2, 5]. Although the feeding preferences of adult *D. speciosa* are well studied under controlled conditions (laboratory experiments and greenhouse), field studies investigating the foraging behaviour of *D. speciosa* in different host plants are lacking.

The feeding preferences of *D. speciosa* constitute a parent-offspring conflict, as female adults need to choose between maximising their reproductive output or the development and growth of their larval offspring. Understanding this conflict could support farmers in the development of pest management strategies to reduce the density of *D. speciosa* in crop fields.

Field studies have investigated the spatial distribution of *D. speciosa* in a single crop, such as tobacco and corn [3, 6], but they did not consider the interactions of the insect with different host plants during its life span and how it affects insect foraging. In order to fill this gap, computational models may provide useful and reliable insights by simulating field conditions and testing different scenarios, when resources and time to accomplish field studies are scarce [7]. The demand for theoretical studies considering the landscape structure has led entomologists to use spatially structured models such as cellular automata (CA). These discrete models represent space as a grid of cells that can assume a finite number of states. Changes in the cell states are given by a set of rules that mimics the insect development and its interaction with the surrounding environment. This has proven to be an efficient tool to describe insect dynamics [8].

Using cellular automata models, [9] showed that the different feeding preferences of *D. speciosa* according to the life stage can be used for insect management, designing crop spatial arrangements in order to reduce insect populations without increasing the use of insecticides. They used a CA model to investigate combinations of different host plants (corn, bean, soybean and potato) and their effects on insect population dynamics. Through these simulations, they found that intercropping corn with any other crop system tested could reduce the spread of the insect through the landscape. However, they did not consider the insects’ decision in moving toward a particular host plant, but rather assumed that the insects move randomly. Also, insect presence and absence were modeled without considering a carrying capacity for adults or immatures. For the genus *Diabrotica*, foraging behaviour is an important characteristic to be investigated in spatial studies, since field results indicate that the insect movement is not random [10]. Additionally, because of the different preferences of each life stage, adults have the challenge to optimise their foraging time before switching crop in order to benefit both stages, immature and adult [11].

With this in mind, the current study used an individual-based model to investigate the influence of the parent-offspring conflict on the population dynamics of *D. speciosa* and the possible consequences for its management. We decided to use this computational approach because it has been successfully used to simulate spatial dynamics of insect pests [8, 9], providing a more realistic scenario by focusing on individual characteristics of each individual of a population [12].

First, we designed an intercropping system, static, composed of corn (favourable for the offspring) and soybean (favourable for the adults), and simulated the insect movement using biological parameters from the literature as model input. Then, we simulated classic types of basic movement modelling, considering a continuous oviposition to simplify the process. Three hypothetical types of adult movement were compared in the proposed area: (a-1) diffusion, (a-2) attracted to a fixed host and (a-3) alternating the preference between hosts. In a more realistic scenario, we considered a pre-oviposition period and compared two distinct strategies common in nature: “mother knows best” (b-1), i.e. adult females foraging for corn during the oviposition period, and “optimal bad motherhood” (b-2), i.e. adult females remain foraging for soybean to maximise their own fitness, using the biological parameters of *D. speciosa*.

Second, using the movement a-3 and a landscape scenario, we focused on studying the foraging behaviour when the insect is allowed to move through multiple areas that change over time in a dynamic landscape based on a crop calendar from the state of São Paulo, Brazil, composed of soybean, corn and fallow areas. This study can provide important information about the effect of this conflict on the spatial dynamics of *D. speciosa* and help in developing plans for integrated management of this pest.

## Methods

A stochastic individual-based model was developed to explore the spatio-temporal behaviour of the insect pest *Diabrotica speciosa*, in two scenarios: (i) *intercropping scenario:* defined as a static landscape composed of soybean and corn arranged in alternate rows; (ii) *landscape scenario*: defined as a dynamic landscape composed of soybean, corn, and fallow areas arranged in blocks that simulate a crop calendar from the state of São Paulo, Brazil.

Insect life span was divided into two main stages: immature (egg, larvae and pupae) and adult. Demographic parameters, including mortality, fecundity and dispersal were dependent on the interaction between insect stage and host type [7, 9]. Immature dispersal was considered as absent, and adult dispersal was varied, considering either a random movement (diffusion) or a directional movement (taxis). For insects transiting between different crops, the values of the demographic parameters were weighted proportionally to the time that the insect spent on each crop.

We obtained data on mortality, development and oviposition rate of the insect pest *D. speciosa* when feeding on corn or soybean (Table 1). The experiments were carried out under controlled conditions (25 ± 2^∘^C; 60 ±10% RH; and photophase of 14h) [2, 5] and the parameters were estimated according to [9]. Regarding the fallow areas, we considered that the insect does not lay eggs or feed in this part of the land. We assumed a sex ratio equal to 1:1 [2, 5].

**Table 1:** Biological parameters obtained from [9]. *ϕ* – oviposition, *γ* – adult mortality (only for adults), *μ*– immature mortality, *σ* – metamorphosis (only for immatures). The unit of all parameters is *day*^−1^

	*ϕ*	*μ*	*σ*	*γ*	PO**	O***
Corn	0.011	0.011	0.040	0.031	10	12
Soybean	0.056	0.045	0.037	0.020	8	30
Fallow	0	1	0	0*		

*We assumed that insects are not able to feed in these areas

**PO: pre-oviposition period (days)

***O: oviposition period (days)

### Algorithm structure

Individual insects and hosts were defined spatially in a bidimensional lattice *L*×*L* with their position given by coordinates (*i*,*j*) where *i*,*j*=1,2,...,*L*. Therefore, in each lattice position (*i*,*j*), the number of insects in each stage and the host type is given. The maximum number of adult and immature insects at each lattice position are, respectively, *n*_*a*_ and *n*_*i*_. These two parameters mimic the environmental carrying capacity. Furthermore, the adult insect carries a counter (*t*_*host*_) that is set to zero when it emerges and increased by one unit for each day that it feeds on a specific host, *t*_*s*_ indicates how many days the adult insect fed on soybean crop, and *t*_*c*_ indicates how many days it fed on corn crop. Each time step corresponds to one day, and each lattice position (cell) represents 1×1 m^2^ of the crop system [9]. One time step involves two processes: (1) birth, development and death associated with the insect life cycle and (2) dispersal of adult insects. All cells in the lattice (the maximum number of insects in the lattice is given by (*n*_*a*_+*n*_*i*_)×(*L*×*L*)) are updated sequentially and periodic boundary conditions are used. The state of the cells changes according to:

I *Insect life cycle*
  1. *Occupied cells.* Each cell of the lattice can be occupied by immature insects, adults or both stages
  - Immatures. Each lattice cell can be occupied by *k*_*i*_ immature insects, where *k*_*i*_∈[0,*n*_*i*_] and *n*_*i*_ is the immature carrying capacity. On each day (*Δt*=1), an immature insect has probability *μΔt* of dying, and *σΔt* of emerging as an adult. Both parameters depend on the host used as the food resource, *p*, located in the cell, therefore *μ*:=*μ*(*p*) and *σ*:=*σ*(*p*). If one of the two events occurs, *k*_*i*_ decreases one unit; when *k*_*i*_=0, the cell becomes empty of immature insects. This rule is applied to all *k*_*i*_ insects found in the selected cell. For each immature that emerges as an adult, the number *k*_*a*_ of adult insects increases by one unit until the cell reaches its carrying capacity, given by *n*_*a*_ (*k*_*a*_∈[ 0,*n*_*a*_]).
  - Adults. Each lattice cell can be occupied by *k*_*a*_ adult insects, where *k*_*a*_∈[ 0,*n*_*a*_] and *n*_*a*_ is the adult carrying capacity. At each time step, an adult insect has probability *γΔt* of dying due to adult insect mortality. If this occurs, *k*_*a*_ decreases one unit; when *k*_*a*_=0, the corresponding cell becomes empty of adult insects. Adult mortality is also a function of the relative time spent between the two crop types; and *γ* is given by:
    $$\gamma := \frac{\gamma_{s}t_{s} + \gamma_{c}t_{c}}{t_{s}+t_{c}} $$
    where *t*_*s*_ and *t*_*c*_ are the times spent at resource S (soybean) and C (corn), respectively, during the adult stage.
  (I-2) *Empty cells.* An empty cell has probability *ϕΔt* of becoming occupied due to oviposition of each insect dwelling in the Moore neighborhood of radius two of the lattice cell plus the cell itself (a Moore neighbourhood of radius R around a cell is the square consisting of all cells not farther than R units from the central cell in any direction). The probability *ϕ* is also a function of the relative time spent between the two crop types:
    $$\phi := \frac{\phi_{s}t_{s} + \phi_{c}t_{c}}{t_{s}+t_{c}} $$
    where *t*_*s*_ and *t*_*c*_ are the times spent at resource S (soybean) and C (corn), respectively, during the adult stage. For each time step, it was assumed that 70% of adults, randomly sorted, are sexually mature, and thus can lay eggs (this percentage can be varied without changing qualitatively the results). Therefore, if the total number of insects in the lattice is, for example, 10, only 7 insects will lay eggs. If oviposition occurs in a particular cell, *k*_*i*_ increases by one.
II *Adult dispersal*
  The velocity of adult dispersal is modified by two parameters, *D* and *f*_*D*_, respectively, the number of dispersal steps, and the proportion of insects dispersing. For instance, *D*=2 means that the insect performs the movement twice in each time step; and *f*_*D*_=0.7 means that 70% of the adults are dispersing. Two situations were modeled: a) adult females are able to lay eggs uninterruptedly after emergence; b) there is a pre-oviposition period, in which adult females do not lay eggs. Based on situations (a) and (b), five types of dispersal were modeled:
  1. Diffusion. It is assumed that dispersal is equally probable in all directions; therefore, *f*_*D*_×*k*_*a*_ adult insects located at the selected site (*i*,*j*) can move to any empty cell (randomly chosen) in its Moore neighborhood of radius one.
  2. Movement to crop type *p* with *p* fixed. Dispersal is driven by the resource type. If the adult insect is feeding on resource *p* it can move randomly (Moore neighborhood of radius one) to another site of the lattice filled with resource type *p*. Otherwise, if the adult insect is not feeding on resource *p*, it explores the environment for *p* in its Moore neighborhood of radius *R*, moving at least one cell in the randomly chosen direction (Moore neighborhood of radius one). If *p* is not found inside *R*, the adult insect moves by diffusion. Insects can move only to sites in the lattice where *k*_*a*_<*n*_*a*_. The rule is applied to all *f*_*D*_×*k*_*a*_ adult insects located in the selected lattice cell.
  3. Movement to crop type *p* with *p* changing. This type follows the same rules described above, but in this case the resource that drives insect movement changes periodically between the crops available in the landscape with frequency $\frac {1}{\tau }$, where *τ* is the time in days spent on resource *p* (the foraging time before switching crop) before changing to the next host. In this case, soybean was chosen as the first target host. After a period of time *τ*, corn was selected as the target host and the process was repeated over time.
  4. “Mother knows best” principle. We assumed that during the pre-oviposition stage, adult females forage for a specific crop (soybean) to fulfill their nutritional requirements. However, once nutrients are acquired and ovary development occurs, adult females will forage for another type of crop (corn) to secure the highest offspring survivorship during the oviposition period. The number of insects dispersing is given by *f*_*D*_×*k*_*a*_, where *k*_*a*_ is the number of adult insects located at the selected site (*i*,*j*). It is important to note that, for movement types (b-1) and (b-2), a counter is added to newly emerged adults to count their physiological age.
  5. “Optimal bad motherhood” principle. This movement is similar in all aspects to (b-1) except that adult females remain foraging for soybean to maximise their own fitness even during the oviposition period.

### Scenarios

#### *Intercropping scenario*

All simulations started with adults (two per cell) in a region with size 20×20 cells in the centre of the lattice. At each site occupied by adults, we assumed that only 70% are able to disperse (*f*_*D*_=0.7) [9]. The first scenario was composed of two different crops, corn and soybean, constant over the time period, arranged in alternating strips measuring *L*×*L*_1_, where *L*_1_ is the width of the crop row. The default set of parameters used was *L*×*L*=600×600, *R*=4, *D*=2, and *n*_*i*_=*n*_*a*_=10. For this scenario, we tested all types of movement described previously. When adults alternate between two crops (movement type a-3), we also evaluated the influence of different values of *L*_1_ and *n*_*a*_ on the foraging time before switching crop. The results presented for all dispersal types in this crop scenario are the mean values of 20 simulations. The duration of our simulations in this scenario was 400 days (*t*=400), which is large enough to guarantee the visualization of a pattern, but short enough to avoid the population to reach the border of the lattice. However, the duration of our simulations does not affect qualitatively the results.

#### *Landscape scenario*

The second scenario was composed of four crop plots with size 150×150 each, representing corn, soybean, or fallow areas. These plots changed over time according to a crop calendar based on the cropping routine from the state of São Paulo, Brazil, in which we considered that crops are susceptible to insect attack during seven continuous months of one year (after removing periods of harvesting, germination, and when plants had not reached a sufficient size to be attacked). The landscape structure was divided into two periods: the first three months (four possible configurations) and the last four months (four possible configurations). The default set of parameters used was *L*=400×400, *τ*=12, *R*=4, *D*=2, and *n*_*i*_=*n*_*a*_=10. For this particular scenario, we only considered the movement type a-3 described previously. We measured the total number of insects and also the size of the area occupied by the population at the end of the simulation (area of the circle with radius given by the root of the mean squared displacement). Density is defined as the ratio between these two measures [9]. The results presented for this scenario are the mean values of 100 simulations. In this study, we considered that the insect dynamics depended only on the landscape type, not considering other abiotic variables, since our interest here was to investigate specifically the effect of the crop arrangement on the behaviour of *D. speciosa*.

## Results

### *Intercropping scenario*

**Fig. 1:**
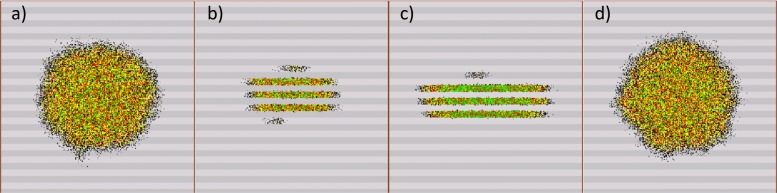
Spatio-temporal distributions of the adult insects at *t*=200. Light-grey and dark-grey rows represent corn and soybean respectively. The landscape is composed of soybean and corn crops arranged in rows of the same size. **a** diffusion pattern observed when the insect moves randomly inside the lattice (movement type a-1); **b** insect distribution considering a movement towards corn (movement type a-2); **c** insect distribution considering a movement towards soybean (movement type a-2); and **d** insect distribution when the direction of movement is periodically changed between soybean and corn with frequency $\frac {1}{\tau }$ (movement type a-3). The density of adults in each cell is represented according to the percentage of the adult carrying capacity used: green >90*%*, red (50*%*,90*%*], yellow (20*%*,50*%*], and black ≤20*%*

Figure 1 shows the spatio-temporal distribution of the adult population for the three movement types when the oviposition is continuous. In (a), (b), (c) and (d), we observe, respectively, diffusion (a-1), movement towards corn (a-2), movement towards soybean (a-2) and periodic alternance of hosts between soybean and corn (a-3). The different colours are related to the density of adults in each cell of the lattice.

**Fig. 2:**
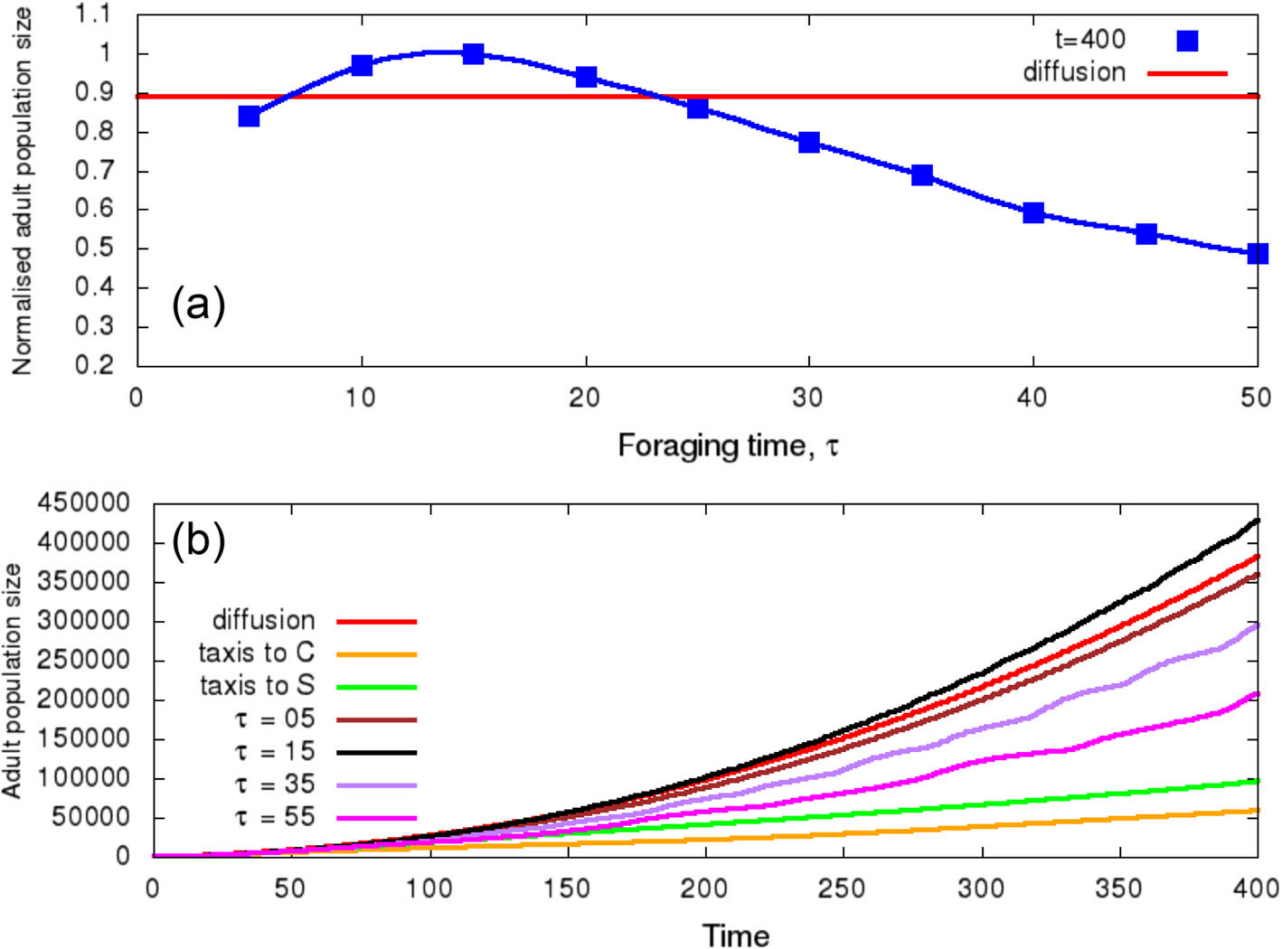
Effect of different types of movement on the dynamics of adult insects, considering a continuous oviposition. **a** The figure shows the evolution of the normalised adult population at *t*=400 for different values of *τ* (movement type a-3). The continuous red line is associated with diffusion (movement type a-1). **b** The figure shows the dynamics of the adult population size over time for different types of movement; the continuous red line is associated with diffusion (movement type a-1), and the orange and green lines are associated with directional movement to corn (C) and soybean (S), respectively (movement type a-2). The other lines are associated with the alternating preference between available hosts for different values of *τ* (moviment type a-3)

Figure 2 shows the min-max normalization of the population size for the three movement types when the oviposition is continuous. We are also able to observe the effect of different values of *τ* (depicted by a blue line with square markers) on the normalised population density of adults at the end of the simulation (*t*=400) for the movement type a-3. When the values *τ* are varied, a maximum number of adults is observed for *τ* between 10 and 20, indicating an optimal value. Figure 2 also shows the corresponding populations dynamics over time. Comparing the 3 types of movement tested, movement a-2 was the worst for the insect population. Regarding the comparison between movement a-1 and a-3, only a foraging time before switching crop (*τ*) close to optimal allows movement type a-3 to outperform random dispersion.

**Fig. 3:**
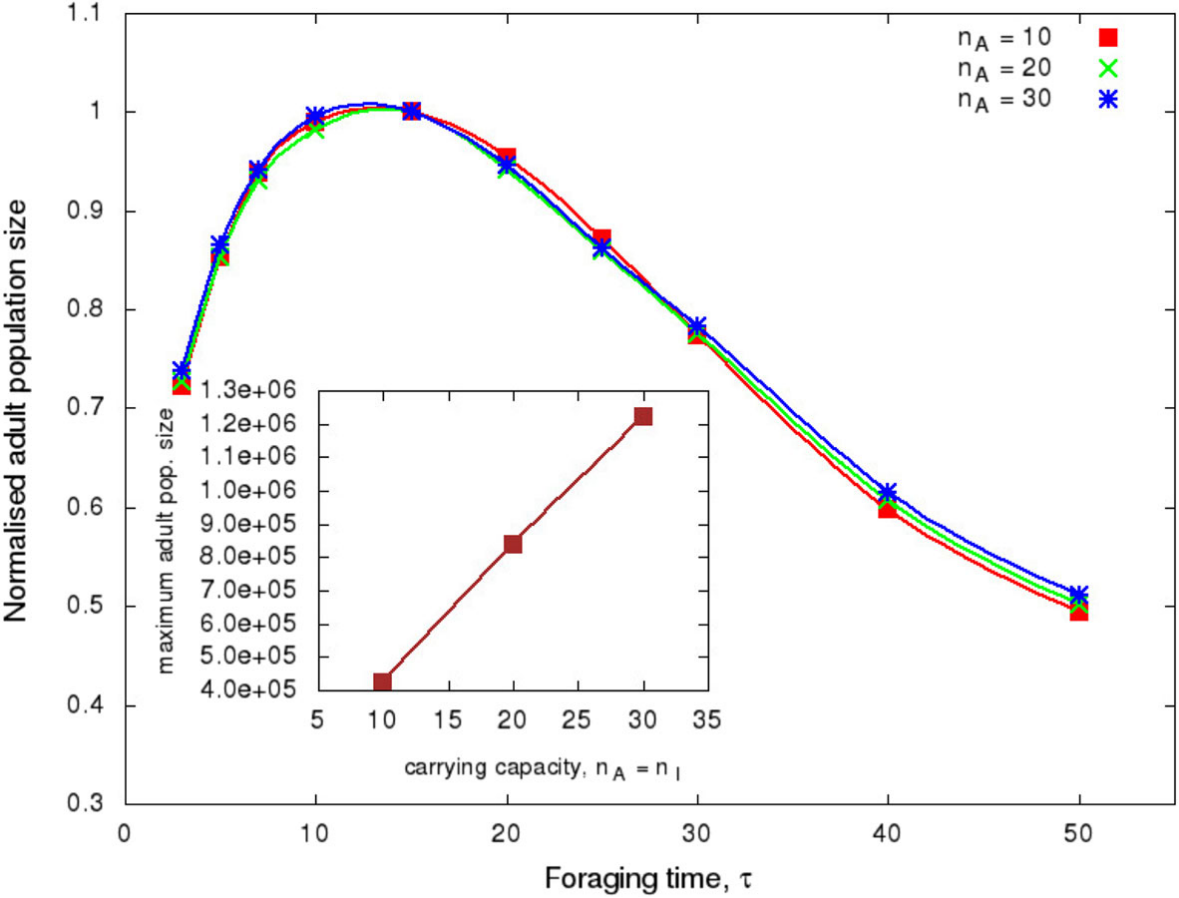
Sensitivity of the parameter *τ* to model parameter *n*_*a*_. Adult and immature carrying capacities were chosen to be the same, *n*_*a*_=*n*_*i*_. The figure shows the normalised adult population size as a function of *τ* for different carrying capacities of hosts with *n*_*a*_ varying from 10 to 30

Given the importance of the foraging period, we decided to determine the sensitivity of this value to other parameters of our model. In Fig. 3, the colours represent the values of different carrying capacities *n*_*a*_, maintaining the values of other parameters constant. It is seen that *τ* does not depend on *n*_*a*_; on the other hand, the total population size increases linearly with *n*_*a*_.

**Fig. 4:**
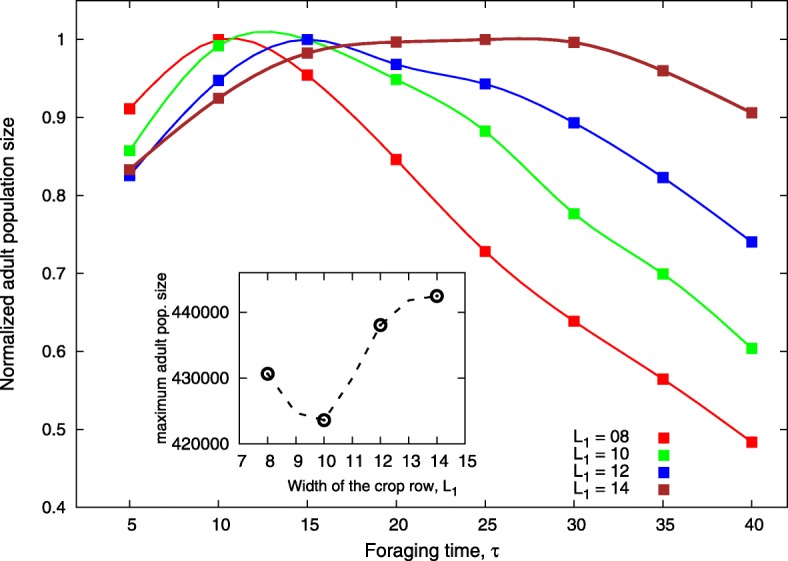
Sensitivity of the parameter *τ* to model parameter *L*_1_. The figure shows the normalised adult population size as a function of *τ* for different widths of crop rows of hosts *L*×*L*_1_ with *L*=600 and *L*_1_ varying from 8 to 14

In Fig. 4, the carrying capacity is fixed at *n*_*a*_=*n*_*i*_=10, and the colours represent different widths of crop rows. It can be seen that the increase in *L*_1_, the width of the crop row, leads to an increase in the foraging time before switching crop that maximises the number of individuals and the curve becomes flatter. We also observed that the population size reached a minimum around *L*_1_=10, increasing monotonically after this point (Fig. 4).

**Fig. 5:**
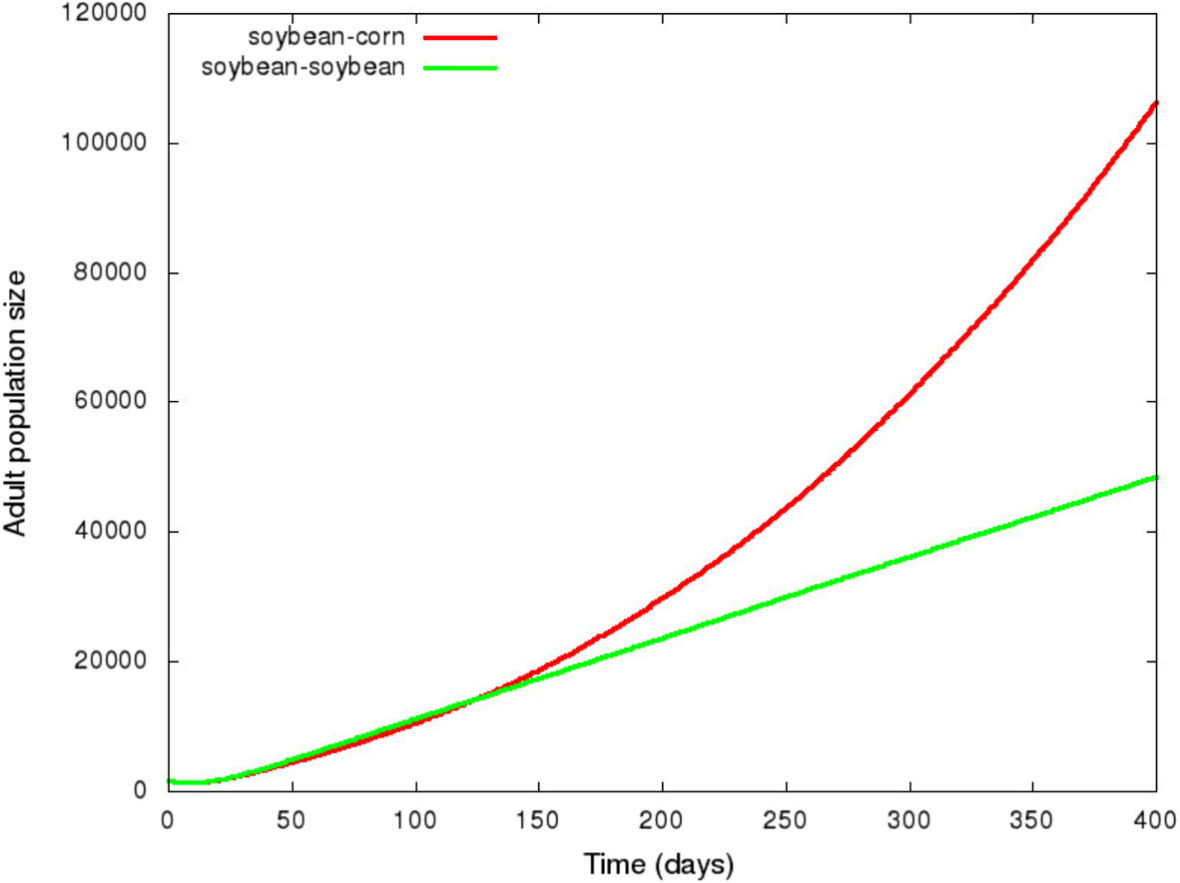
Evolution of the adult population over time for two different principles. In green, adults are remaining in the same crop over the entire stage (bad optimal motherhood). In red, adults search for a crop to maximise the survival and growth of their offspring during the oviposition period (mother knows best)

In Fig. 5, we compared movement types b-1 and b-2, when the pre-oviposition and oviposition periods are simulated. We observed that the “mother knows better” principle (females foraging for corn to maximise their offspring survival during the oviposition period) is preferable for the population growth to the “optimal bad motherhood” principle (females remaining on soybean).

### *Landscape scenario*

**Fig. 6:**
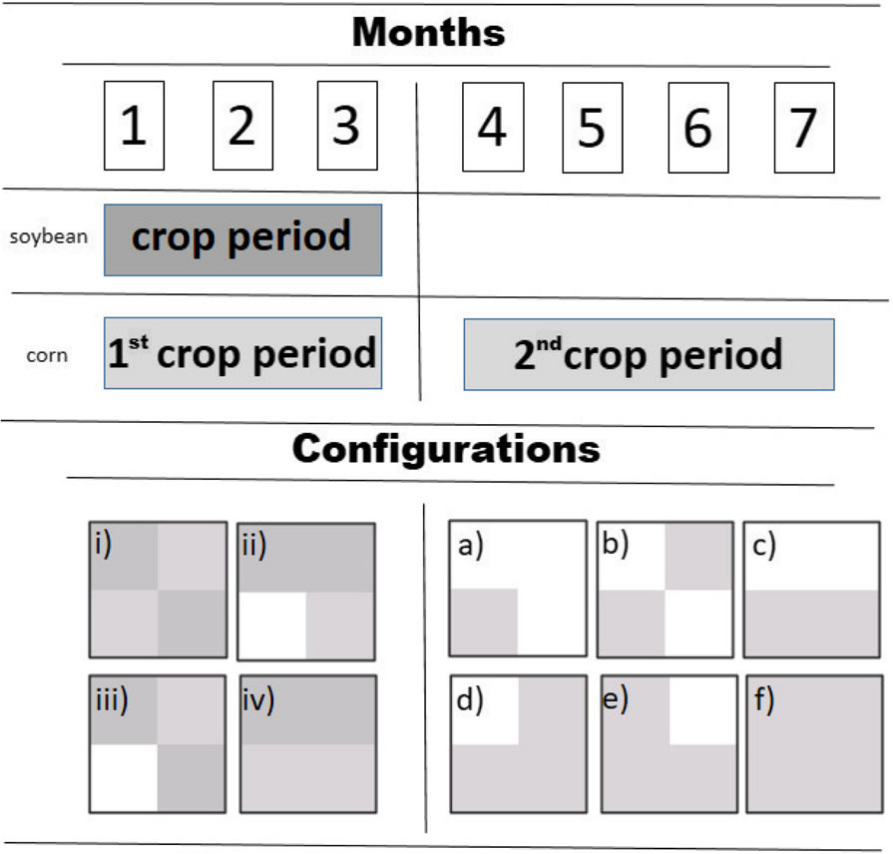
Crop calendar indicating the crop periods of corn and soybean considered in the current study. Each month represents a period of 30 days (time steps). The figure also presents all configurations simulated during the first three months (numbers) and last four months (letters). Dark grey – Soybean; Light Grey – Corn; White – absence of crops (fallow areas). Figures (i), (ii), (iii) and (iv) represent the four configurations used during the first three months; the next six configurations used in the last four months are indicated by a, b, c, d, e, and f

**Fig. 7:**
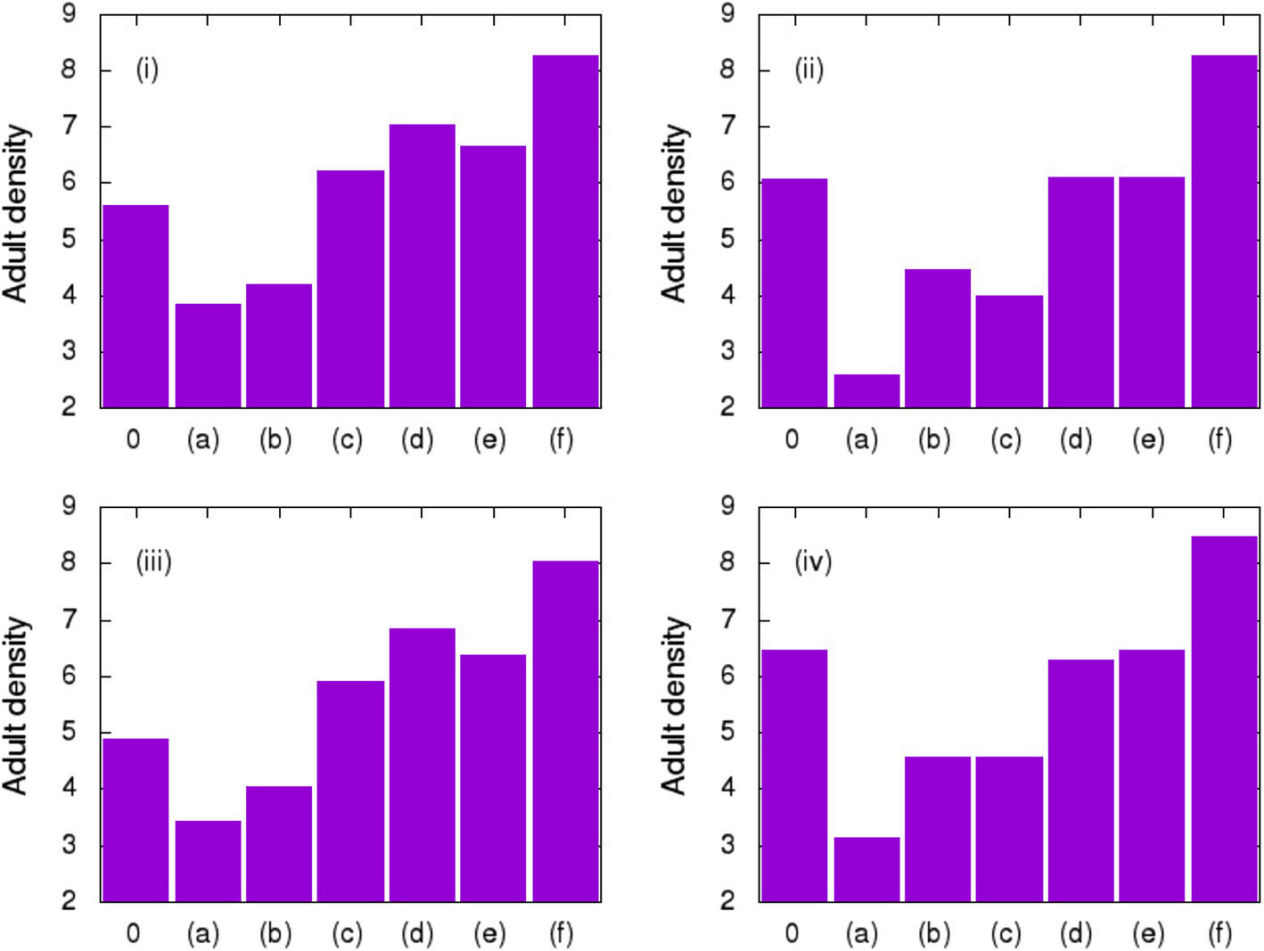
Adult density in the lattice area in each of the dynamic landscapes simulated according to designated configurations. Numbers (i), (ii), (iii) and (iv) correspond to the four scenarios simulated during the first three months, and the letters (a)-(f) to the six configurations of the landscape assumed for the next four months

Figure 6 shows the crop calendar adopted in the dynamic landscape. The four configurations, (i) - (iv), were used during the first three months, and the six last configurations, (a) - (f), were used in the last four months. Using the movement type a-3 and fixing *τ*=12, the population densities of adults at *t*=90 (the end of the first period) and *t*=210 (the end of the second period) were measured (Fig. 7). Each of the four figures (i), (ii), (iii) and (iv) represents the four configurations used during the first three months. The columns in each of these graphs represents the six subsequent configurations adopted in the last four months (a, b, c, d, e, f); and 0, when only the first period is considered.

We observed that arrangements with larger continuous blocks of soybean (Fig. 7, scenarios ii and iv – 0) had higher numbers of insects than the more heterogeneous areas (Fig. 7, scenarios i and iii – 0) that alternated blocks of different crops. The number of fallow areas in the landscape was another important factor, implying a smaller number of insects, because of the reduction of available hosts. In the succeeding four months (second period), increasing the number of fallow areas reduced the number of adult insects, independently of the configuration adopted during the first period. Similarly to the first three months, we also observed different densities in landscapes that adopted the same number of fallow areas (Fig. 7, configurations b and c) but arranged in different positions (heterogeneity).

## Discussion

### *Intercropping scenario*

Since larval stages have limited mobility, they feed on the host plant selected by their mothers. In many cases, host preference of adult insects is correlated with their oviposition preferences [11, 13]. However, for certain insect species, such as *D. speciosa*, this behaviour tends to be more complex because hosts that maximise offspring development also reduce the reproductive potential and survival of the adults [13]. This creates a parent-offspring conflict, in which females need to choose between laying eggs on the host plant that maximises the offspring survival (mother knows best) or remaining on the host plant that increases its own longevity (optimal bad motherhood) [14, 15].

However, Fig. 2 suggests the existence of an optimal period of time (*τ*∼12 days) spent foraging for each host that maximises the population growth (movement type a-3), implying in a balance between the search for soybean to maximise adult longevity and for corn to maximise the offspring survival. We also observed that for cases involving this conflict, oviposition sites should be influenced by the proximity of resources for the adult [16]. In fact, this was observed when we changed the width of the crop strip inside the area (Fig. 4).

In general, the value of *τ* increased and were also broader with crop row width (Fig. 4). Larger rows have a large food supply that supports higher numbers of insects. Since we chose soybean as the first target host, adult insects benefit from staying longer on this host before moving on to corn crops. Additionally, narrow rows require an accurate foraging time before switching crop, sharpening the curve, because of the limited availability of resources, forcing the insect to be more active through the lattice to feed on a particular host. However, we observed that *L*_1_=8 supports higher numbers of insects in the lattice than *L*_1_=10. As observed by [17], the idea that spatial heterogeneity in itself reduces pest population has been inconsistent with field data. In intercropping systems, the rows need to be large enough to subject the insect to spend some time feeding on undesirable hosts when they are foraging for the preferred host [18]. Otherwise, the intercropping system will be as effective as a homogeneous landscape to manage the insect pest [9]. We did not observe an influence of carrying capacity on *τ*, but the increase in *n*_*a*_ leads to an increase in the adult population size for the same reason discussed previously (availability of resources)(Fig. 3).

The existence of a balance between the foraging times spent in each host becomes clear in Fig. 5. When the pre-oviposition period is simulated, we observed that the “mother knows best” principle is preferable for the population growth of this insect pest to the “optimal bad motherhood” principle (Fig. 5). It happens because adult females initially spend the pre-oviposition period on soybean, which provides nutritional advantage for themselves, but choose corn to lay eggs. A similar behavior was also observed in *Cephaloleia* beetles that select native hosts to favour the offspring [19]. However, it is not a rule for herbivore insects. [20] observed that the polyphagous vine weevil (*Otiorhynchus sulcatus*) tend to lay eggs on plants with smaller root systems, which negatively affect offspring performance. According to [21], stronger oviposition preference-offspring performance relationships are more associated to specialist herbivores than generalist species. However, our study brings a different result, which indicates that a “mother knows best” hypothesis is more appropriate for a generalist species (*D. speciosa*). Although well documented for other insect species [22], no field or laboratory experiment has yet been conducted to demonstrate this behaviour for *D. speciosa*.

### Landscape scenario

Regarding the dynamic landscapes, we observed that for the first three months, the major factor influencing the population density was the heterogeneity in the landscape. The number of adults of *D. speciosa* is favoured by a continuous area of soybean, since their survival is maximised by feeding on this host [5]. Habitat management that increases the spatial heterogeneity of crops and avoids creating vast areas of a single crop (monoculture) can reduce the densities of insect pests [23, 24]. When an insect must deal with different crops, it is subjected to different environmental challenges that can be unfavourable for its reproduction or development, which would reduce its fitness [19]. Although the offspring survival is maximised on corn, the female needs to feed on soybean first, otherwise will need to adapt to less suitable host plants [13].

The adoption of a corn fallow area caused a reduction in the number of insects, as expected, since corn is a potential host for adult beetles, although not their preferred host [2, 25]. This is the first study to model this issue, considering the foraging behaviour of *D. speciosa*, and indicated the efficiency of fallow areas in maintaining low densities of this pest. Previously, [9], using an individual-based model, found that heterogeneity in crops could reduce the densities of *D. speciosa*, but did not examine the effect of fallow areas. Soybean fallow areas have been recommended by Embrapa (Brazilian Agriculture Research Corporation) and adopted in Brazil in the last few years in order to control insects and fungi that may impact soybean production negatively, comprising a period between 60 and 90 days, generally over winter and early spring (June - September) [26].

For scenarios i-c and iii-c, when the areas are arranged in two continuous rows, reducing the crop heterogeneity, the number of insects observed is higher than in the other landscapes with the same composition (i and iii-b, respectively). [9] found the same pattern, since it reduces perturbations in insect dynamics, reducing the range of conditions to which the insect is subjected. However, for arrangements ii-c and iv-c, the value was less than or equal to those of ii-b and iv-b, respectively. For both these cases, the initial arrangement of the landscape (first three months) had a continuous row of soybean, where most insects were concentrated, since adults develop better on this host. When this row was completely removed and replaced by fallow areas, the population was more affected than in ii-b and iv-b. The results suggest that alternating different hosts in adjacent crop areas as well as inserting fallow periods in the crop calendar can support the management of this insect pest. We showed that it is possible to grow crops on all four lands and reduce insect density in approximately 70% (Fig. 7, ii-a) by simply leaving them to rest during a fallow period.

## Conclusion

In order to verify the effects of the parent-offspring conflict caused by the different feeding preferences of *Diabrotica speciosa*, we initially tested 3 hypothetical types of movement, considering a continuous oviposition. Our results indicated that the best strategy for the insect consisted in adults alternating the host preference according to a foraging time before switching crop (*τ*). Movement type a-3 allows adults to maximise the development and growth of their larval offspring on corn after increasing their own longevity and reproductive output on soybean during a period *τ*. Such result suggests the existence of an optimal period of time to alternate between each host.

The balance between the foraging for each host was clear when we included a pre-oviposition period in our simulations. The results revealed a better performance of the population growth when adults feed on the most appropriate host for themselves during the pre-oviposition period (soybean) and prioritize the host that provides better conditions for the development and growth of their larval offspring during the oviposition period (corn). It indicates that a “mother knows best” strategy is more suitable for *D. speciosa* than a “optimal bad motherhood”. However, field and laboratory experiments are needed to determine whether the species actually shows this behaviour.

In the second part of our study, we verified that the arrangement of crops plots in a landscape influences the population dynamics of *D. speciosa*, because of the parent-offspring conflict. Large and continuous areas of soybean increases the adult density, because the arrangement provides initially a large supply of the best nutritional source to fulfill the nutritional requirements of adults. Therefore, arranging a more diverse landscape with soybean and corn plots can represent a control strategy since it subjects the adult to different environmental challenges that can be unfavourable for its reproduction or development. Another potential pest management strategy is the use of fallow periods during the year. We showed a strong reduction in the number of insects in landscapes that adopted such approach.

Finally, we would like to mention the advantages of using a computational model. Our study highlights the benefits of computational modelling which, compared to experimentation involving landscape arrangements, is less costly, more flexible and easier to replicate. Modelling approaches present great potential to explore other case studies involving movement ecology and insect behaviour. However, we did not consider the density dependence in movement or feeding choices and it remains a limitation to be overcome in future works.

## Data Availability

Not applicable.

## Abbreviations

CA: Cellular Automata
Embrapa: Brazilian Agriculture Research Corporation
